# A novel glycosyltransferase-related lncRNA signature correlates with lung adenocarcinoma prognosis

**DOI:** 10.3389/fonc.2022.950783

**Published:** 2022-08-18

**Authors:** Chengyu Bian, Xinti Sun, Jingjing Huang, Wenhao Zhang, Guang Mu, Ke Wei, Liang Chen, Yang Xia, Jun Wang

**Affiliations:** ^1^ Department of Thoracic Surgery, Jiangsu Province People’s Hospital and the First Affiliated Hospital of Nanjing Medical University, Nanjing, China; ^2^ Department of Thoracic Surgery, Tianjin Medical University General Hospital, Tianjin, China

**Keywords:** Lung adenocarcinoma, glycosyltransferase, lncRNA, prognosis, biomarker

## Abstract

**Background:**

Lung adenocarcinoma (LUAD) is one of the most fatal cancers in the world. Previous studies have shown the increase in glycosylation level, and abnormal expressions of related enzymes are closely related to various cancers. Long non-coding RNAs (lncRNAs) play an important role in the proliferation, metabolism, and migration of cancer cells, but the underlying role of glycosyltransferase (GT)-related lncRNAs in LUAD remains to be elucidated.

**Methods:**

We abstracted 14,056 lncRNAs from The Cancer Genome Atlas (TCGA) dataset and 257 GT-related genes from the Gene Set Enrichment Analysis (GSEA) database. Univariate, LASSO-penalized, and multivariate Cox regression analyses were conducted to construct a GT-related lncRNA prognosis model.

**Results:**

A total of 2,726 GT-related lncRNAs were identified through Pearson’s correlation analysis, and eight of them were utilized to construct a GT-related lncRNA model. The overall survival (OS) of the low-risk group continued to be superior to that of the high-risk group according to the subgroups classified by clinical features. The risk model was proved to have independent prognostic characteristics for LUAD by univariate and multivariate Cox regression analyses. The status of the tumor immune microenvironment and the relevant immunotherapy response was significantly different between the two risk groups. The candidate drugs aimed at LUAD subtype differentiation were identified.

**Conclusion:**

We constructed a risk model comprising eight GT-related lncRNAs which was identified as an independent predictor of prognoses to predict patient survival and guide-related treatments for patients with LUAD.

## Introduction

Lung cancer is the leading cause of cancer-related deaths worldwide, accounting for 18.0% of all cancer deaths, and lung adenocarcinoma (LUAD) is the most abundant subtype of lung cancer (1–3). Data reported by the China National Cancer Center indicated that lung cancer accounts for 24.6% of new cancer cases in men and 15.2% of new cancer cases in women, respectively (4). Even with timely interventions such as surgery, chemotherapy, radiotherapy, and new targeted therapies, LUAD remains one of the deadliest diseases (5–7). The main reason for poor prognosis is the lack of effective diagnostic methods for cancer occurrence and recurrence. Therefore, the search for more effective prognostic biomarkers for LUAD is imminent.

LncRNA is a subtype of RNA with more than 200 nucleotides in length (8). By interacting with other biological molecules, such as miRNAs, mRNAs, transcription factors, and RNA-binding proteins, they can accomplish numerous biological functions such as tumorigenesis and apoptosis (9, 10). For example, lncRNAs may be able to exert cytoprotective activity by modulating the transcription and translation of mRNAs (11). Numerous emerging studies suggested that lncRNAs were involved in the regulation of cell function, in both normal and abnormal states (12). Luo et al. revealed that lncRNA GAS6-AS1 could inhibit progression as well as glucose metabolism reprogramming by repressing the E2F1-mediated transcription of GLUT1 in LUAD (13). Zhen et al. reported that lncRNA DANCR was upregulated and miR-216a expression was negatively correlated with lncRNA DANCR expression in LUAD, which suggested that lncRNA DANCR may promote lung cancer by binding to miR-216a (14). It has been demonstrated that the dysfunction of lncRNAs can have a detrimental effect on cellular processes, including tumor cell proliferation, invasion, and apoptosis, which results in a poor prognosis (15). However, the role that lncRNAs play in the occurrence and prognosis of LUAD has yet to be systematically analyzed.

Glycosylation is an abundant and diverse posttranslational modification of proteins that occurs on all eukaryotic cells (16). Glycosylation plays a crucial role in cellular adhesion and stability, as well as intercellular communication (17). Studies showed that elevated levels of glycosylation, including the abnormal expression of related proteins, have an important relationship with various cancers (18, 19). Researchers have shown that abnormal tumor glycosylation alters the perception of the tumor by the immune system and can also activate immunosuppressive signals through the glycan-binding receptors. Consequently, glycan signatures found on tumor cells can be considered to be a novel type of immune checkpoint (20). It has been reported that the genes involved in mucin O-glycosylation in LUAD were significantly upregulated, and the immune response in lung squamous cell carcinoma (LUSC) was blocked, indicating that abnormal glycosylation might lead to the biogenesis and progression of non-small cell lung cancer (NSCLC) (21).

In our study, we obtained 2,726 GT-related lncRNAs through Pearson’s correlation analysis. Then a GT-related lncRNA prognosis model was established for accurate OS prediction of patients with LUAD by univariate, LASSO, and multivariate Cox regression analyses. Next, the prognosis and clinical characteristics of the two risk groups were analyzed based on our model. Additionally, we explored the relationship association with the tumor immune microenvironment and immunotherapy responses. Overall, the results of this study provide a fresh perspective and insights regarding potential strategies for the treatment of LUAD patients.

## Methods

### Data acquisition

All data of the LUAD patients (normal = 59 and tumor = 535) were downloaded from TCGA database (https://portal.gdc.cancer.gov/repository). Based on the lncRNA annotation file obtained from the GENCODE (https://www.gencodegenes.org/human/) website and Ensemble IDs, we annotated 14,056 lncRNAs in TCGA dataset (**Supplementary Table S1**).

### Acquisition of the GT-related genes

Two hundred fifty-seven GT-related genes (**Supplementary Table S2**) were downloaded from the GSEA database (http://www.gsea-msigdb.org/gsea/msigdb/search.jsp).

### Screening the GT-related lncRNAs

A total of 2,726 GT-related lncRNAs were identified using Pearson’s correlation analysis with R (version 4.1.2) according to the standard of |Pearson R| >0.4 and P<0.001.

### Construction and validation of the GT-related lncRNA risk score model

To make the analysis more accurate, LUAD patients whose OS value was missing or shorter than 30 days were removed. Four hundred ninety LUAD samples were used for subsequent analysis, and they were distributed into training set (n = 246) and testing set (n = 244) at random. The risk model associated with GT-related lncRNAs was constructed using the training set and validated by the testing set. **Supplementary Table S3** shows the baseline characteristics of these two sets. Utilizing R package “glmnet”, we conducted univariate Cox, LASSO, and multivariate regression Cox analyses to construct our model (22). The risk score with formula was as follows:


$$Risk\;score=\sum_{k=1}^{n}Coef(lncRNA)*expr(lncRNA^{k})$$

The coef (lncRNA) represents the correlation coefficient between lncRNAs and survival, and expr represents the expression of lncRNAs. According to the median risk score, patients were divided into high-risk and low-risk groups. We conducted survival analysis for appraising diversities in the OS between the two risk groups with the R packages “survMiner” and “survival”.

### Independence of the GT-related lncRNA model

We further tested the ability of our model to predict OS independently of other clinical features through multivariate and univariate COX regression analyses.

### Nomogram

Using various clinical factors and our risk score, a LUAD prognostic nomogram was developed to estimate the probability of 1-, 3-, and 5-year survival. A calibration plot and the C-index were constructed for evaluating the accuracy and consistency of the nomogram.

### Principal component analysis (PCA) and functional enrichment analysis

PCA was applied to analyze the scatter patterns between the two risk groups. We performed GO enrichment analysis and KEGG pathway analysis to investigate the underlying molecular mechanisms of the GT-based model by the R package “clusterProfiler” (23). Additionally, Gene Set Enrichment Analysis (GSEA) was conducted by GSEA software (version 4.2.3) to further investigate the possible enrichment pathways among different risk score groups (24). The mRNA–lncRNA co-expression network was constructed using Cytoscape (version 3.9.1).

### Exploration of tumor immune microenvironment and immunotherapeutic treatment

R package “maftools” was applied for computing the mutation data. Then, R package “ESTIMATE” was utilized to calculate the stromal score, immune score, and estimate score (25). The proportions of 22 tumor-infiltrating immune cell types shown in the bar chart and heatmap plot were identified by the CIBERSORT analytical tool and single-sample gene set enrichment analysis (ssGSEA) algorithm. Additionally, we applied the TIDE algorithm to predict the likelihood of the immunotherapeutic response (26).

### Exploration of potential drugs in clinical treatment

The half-maximal inhibitory concentration (IC_50_) of compounds obtained from the Genomics of Drug Sensitivity in Cancer (GDSC) was calculated using the R package “pRRophetic” in TCGA project of LUAD datasets for potential drugs.

### Chromogenic *in situ* hybridization

A pair of tissue samples embedded by paraffin was deparaffinized in dewaxing transparent liquid and dehydrated by pure ethanol. After the slices were boiled in the retrieval solution for 15 min and naturally cooled, proteinase K working solution was added to cover objectives and incubated at 37°C for 20 min. The sections were further prehybridized for 1 h at 37°C. After hybridization and washing, the hybridization solution containing Imaging Oligo (DIG) and mouse anti-digoxigenin-labeled peroxidase were added successively to incubate. The expression of the eight lncRNAs was further visualized with DAB reagent and hematoxylin. Under a conventional light microscope, the nucleus stained with hematoxylin was blue, and the positive expression of DAB was brownish-yellow. From this, we could know the expression quantity and localization of the eight lncRNAs in LUAD and para-cancerous tissues. Detailed information for the chromogenic *in situ* hybridization (CISH) experiment is provided in **Supplementary Methods**, and probe sequences of those lncRNAs are listed in **Supplementary Table S4**. Additionally, lncLocator 2.0 was used to predict the subcellular distribution of the eight lncRNAs.

### RNA isolation and quantitative real-time PCR

Total RNA of 10 pairs of tissues was extracted with TRIzol reagent. The primer sequence is shown in **Supplementary Table S5**. Levels of the eight lncRNAs were detected by quantitative real-time PCR (qRT-PCR), which were normalized against β-actin RNA by the comparative Ct method. The expression levels were depicted using GraphPad Prism 9 software.

### Statistical analyses

Data were analyzed using the R platform (Version 4.1.2). P< 0.05 was considered statistically significant if no special instructions were given.

## Results

### The source of GT-related lncRNAs

**Figure 1:**
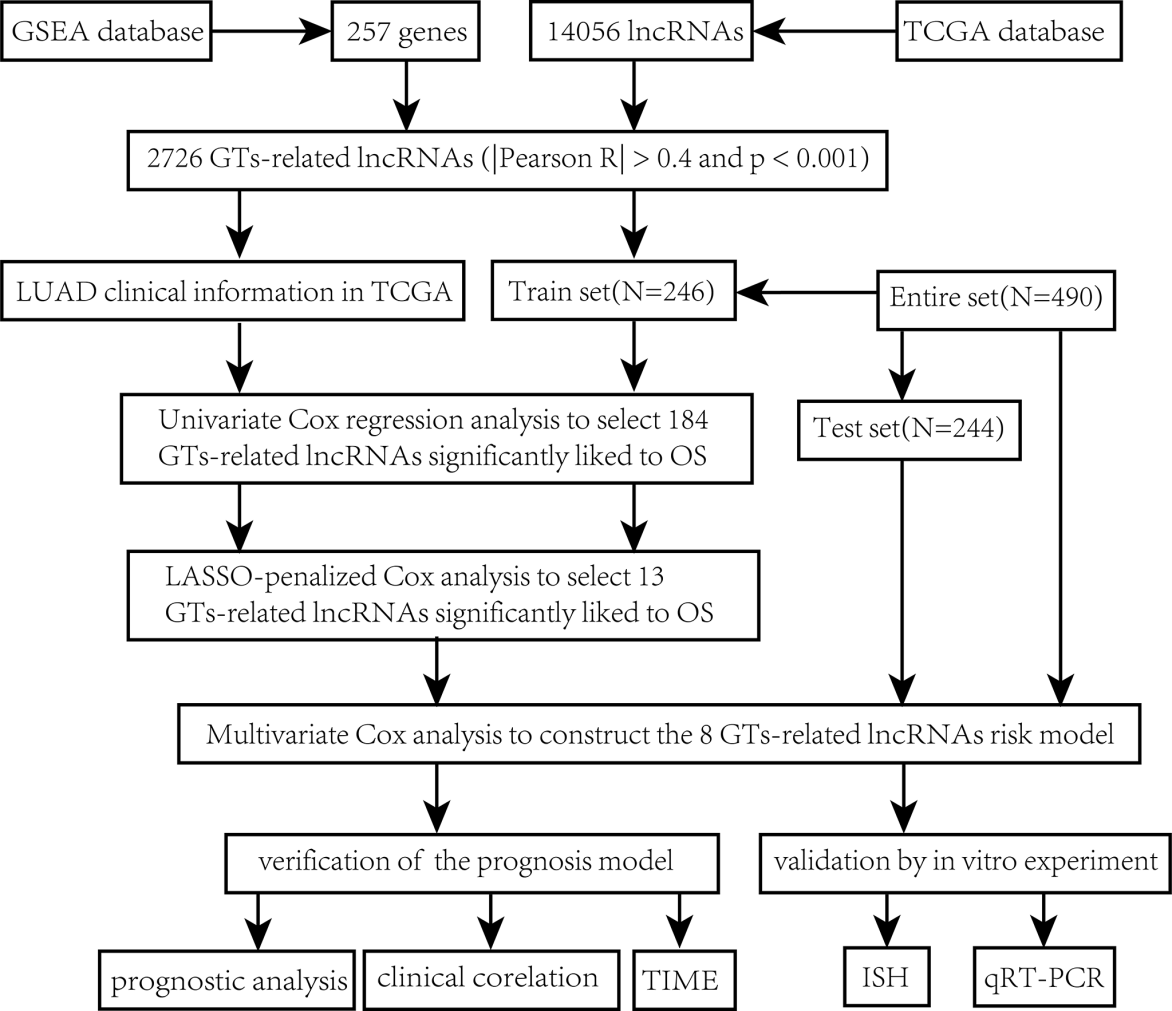
Flowchart.

**Figure 2:**
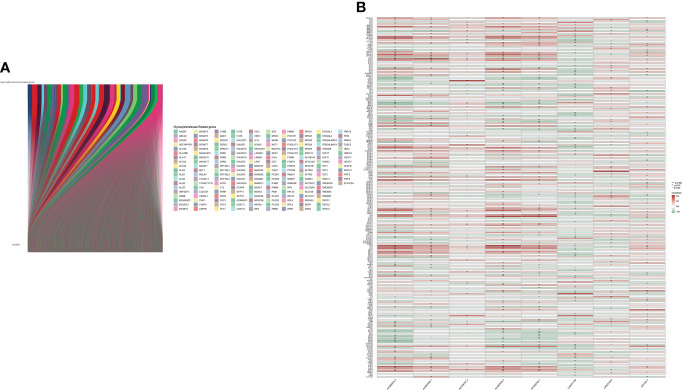
Identification of GT-related lncRNAs. **(A)** The Sankey diagram. **(B)** Heatmap of the correlation between GT-related genes and the eight prognostic GT-related lncRNAs in TCGA entire set.

The main workflow of constructing the risk model and its related analyses are depicted in **Figure 1**. Two hundred fifty-seven GT-related genes and 14,056 lncRNAs were acquired from TCGA database and GSEA database, respectively. GT-related lncRNAs were defined as lncRNAs significantly associated with at least one of the 257 GT-related genes through Pearson’s correlation analysis (|Pearson R| > 0.4 and P< 0.001). Finally, the GT-related lncRNA co-expression network was visualized in the Sankey diagram, in which 2,726 lncRNAs were identified as GT-related lncRNAs (**Figure 2A**). **Figure 2B** demonstrates the correlation between GT-related genes and lncRNAs.

### Construction of a risk model and its related validation

**Figure 3:**
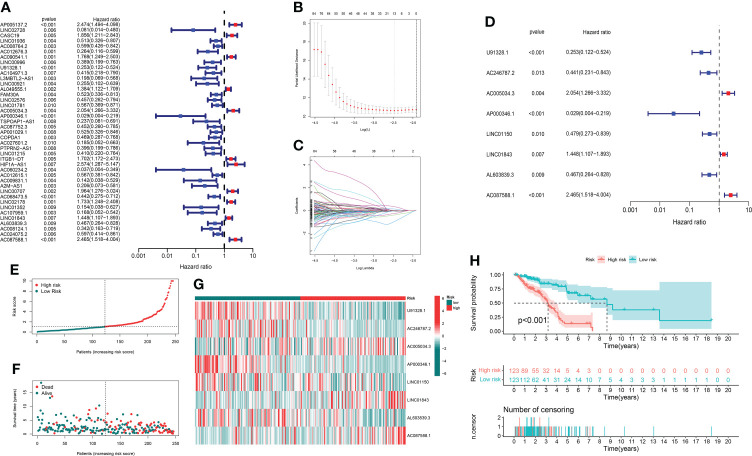
The risk model and its prognostic value in TCGA training set. **(A)** Univariate Cox regression analysis. **(B, C)** The LASSO-penalized Cox analysis. **(D)** Multivariate Cox regression analysis. **(E, F)** Distribution of survival time and risk scores in the training set. **(G)** Clustering analysis heatmap shows the expression of the eight prognostic lncRNAs between the two risk groups in the training set. **(H)** Survival analysis in the training set.

Through univariate Cox regression analysis of 2,726 GT-related lncRNAs in TCGA training set, we found that there was a significant correlation between 184 GT-related lncRNAs and OS (**Supplementary Figure S1**). **Figure 3A** shows the top 30 of the 184 GT-related lncRNAs. LASSO-penalized Cox analysis is a commonly used multiple regression analysis method which could forecast accurately and avoid overfitting to identify predictors and predict clinical outcomes effectively. Hence, 13 GT-related lncRNAs were screened through LASSO-penalized Cox analysis and then used in multivariate analysis for autocephalous prognostic proteins (**Figures 3B, C**). Eight GT-related lncRNAs were discerned as dependent prognostic proteins associated with OS and used to construct the risk model for evaluating the prognostic risk of LUAD patients (**Supplementary Table S6**) (**Figure 3D**). The formula was as follows: risk score = (U91328.1×-1.25527342956323) + (AC246787.2×-0.538805876294878) + (AC005034.3×0.470136953911747) + (AP000346.1×-4.05635656586431) + (LINC01150×-0.589753468090618) + (LINC01843×0.388777844165942) + (AL603839.3×-0.586773218794198) + (AC087588.1×0.952487737586117). LUAD samples are divided into low-risk group and high-risk group according to the median risk score. **Figures 3E, F** depict the risk grade distribution as well as the survival status and time in the two different risk groups, respectively. **Figure 3G** shows the expression level of the eight GT-related lncRNAs in each patient. The OS of the low-risk group was significantly superior in survival analysis (P< 0.001) (**Figure 3H**).

**Figure 4:**
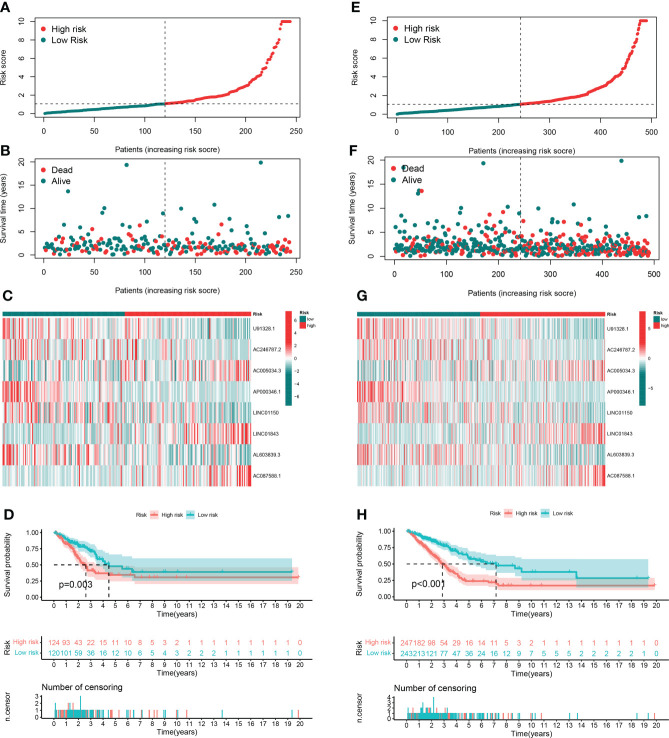
Prognostic value in TCGA testing and entire sets. **(A, B)** Distribution of survival time and risk scores in the testing set. **(C)** The clustering analysis heatmap displayed the expression of the eight prognostic lncRNAs between the two risk groups in the testing set. **(D)** Survival analysis in the testing set. **(E, F)** Distribution of survival time and risk scores in the entire set. **(G)** The clustering analysis heatmap displayed the expression of the eight prognostic lncRNAs between the two risk groups in the entire set. **(H)** Survival analysis in the entire set.

In order to evaluate the prognostic ability of the model, we calculated the risk score using a uniform formula for each patient in the testing set and the entire set. **Figures 4A–C** show the distribution of risk grades, survival status, and time in the testing set between the two different risk groups, as well as the expression of the GT-related lncRNAs. The same content in the entire set is shown in **Figures 4E–G**. Survival analyses of the testing and entire sets demonstrated that the OS of LUAD patients with lower risk scores was superior to that with higher risk scores, which was the same as that of the training set (**Figures 4D–H**).

**Figure 5:**
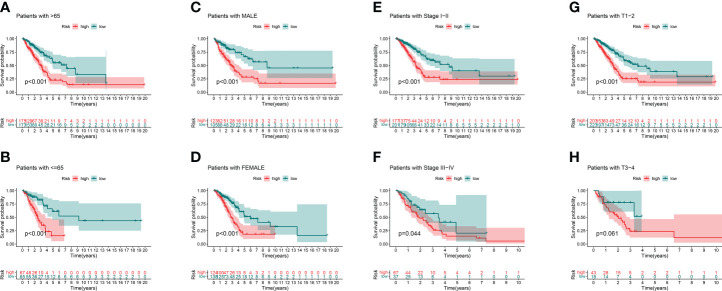
Kaplan–Meier survival curves of clinical stratification of OS between the high- and low-risk groups in TCGA entire set. **(A, B)** age (>65 or ≤65). **(C, D)** Sex (male or female). **(E, F)** TNM stage (I–II or III–IV). **(G, H)** T (T1–2 or T3–4).

We analyzed the OS discrepancies stratified by clinic-pathologic features. The OS of the low-risk group was also superior among subgroups classified by age, sex, TNM stage, or tumor stage (**Figures 5A–H**). The superiority was significant except in the T3–4 subgroups (**Figure 5H**).

### Independence of the model and prognostic nomogram

**Figure 6:**
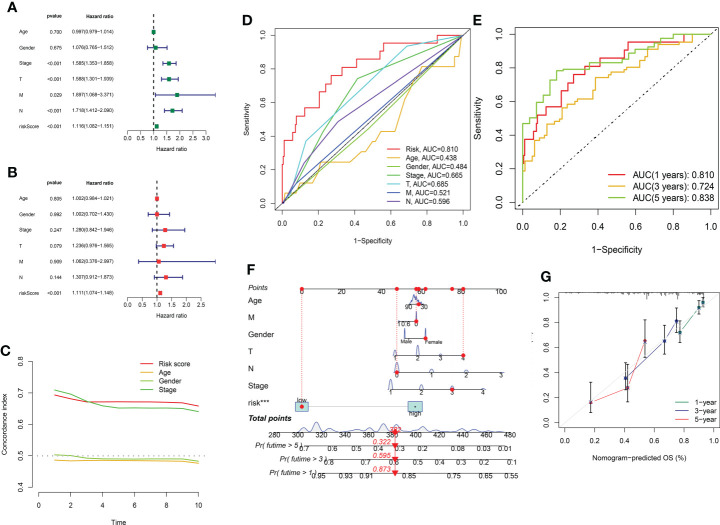
Assessment of the independence of the model and the prognostic nomogram. **(A, B)** Univariate and multivariate analyses of the clinical characteristics and risk score with the OS in TCGA entire set. **(C)** Concordance indexes of the risk score and clinical characteristics in TCGA entire set. **(D, E)** ROC curves of the clinical characteristics and risk score predicting 1-, 3-, and 5-year survival in the training cohort. **(F)** Nomogram. **(G)** Calibration plot.

In order to evaluate the independence of the eight GT-related lncRNA model, we carried out univariate and multivariate Cox regression analyses in TCGA entire set. The HR and 95% CI of the risk score were 1.116 and 1.082–1.151, respectively, in univariate Cox regression analysis (P< 0.001) (**Figure 6A**). **Figure 6B** depicts that the HR was 1.111 and the 95% CI was 1.074–1.148 in multivariate Cox regression analysis (P< 0.001). Thus, we can infer that our risk model has no relationship with gender, age, stage, or other clinicopathological features. In the training cohort, the concordance index of risk score was higher than that of other clinical characteristics with the extension of time, and the AUC of risk grade was also greater than that of other clinicopathological features, which indicates that the risk score could predict the OS of LUAD better and our model was reliable and accurate (**Figures 6C, D**). The ROC curves in the training set and the prognostic nomogram in the entire cohort also confirmed this conclusion (**Figures 6E, F**). The calibration chart forecast the possibility of the 1-, 3-, and 5-year survival in the entire cohort, indicating that the observed ratio is in ideal agreement with the predicted ratio (**Figure 6G**).

### PCA

**Figure 7:**
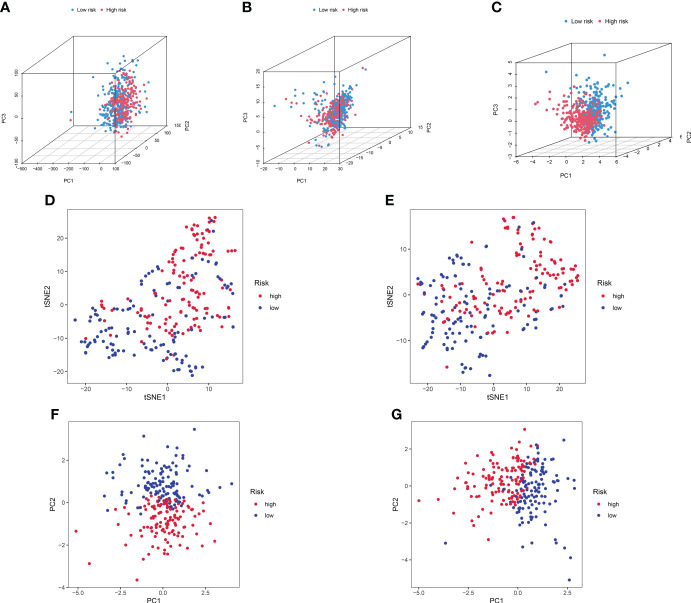
PCA between the high- and low-risk groups based on **(A)** entire gene expression profiles, **(B)** 257 GT-related genes, and **(C)** eight-lncRNA model. **(D, E)** The tSNE analysis between risk groups in the training and test sets. **(F, G)** PCA between risk groups in the training and test sets.

We further conducted PCA based on the whole gene expression data, 257 GT-related genes, and our prognostic model for verifying the discrepancies between the two risk groups (**Figures 7A–C**). The results based on our prognostic model showed that there were significant discrepancies in distribution between the two risk groups, indicating that the grouping ability of our prognostic signature was obviously superior to that of the other two grouping schemes. **Figures 7D–G** shows the tSNE analysis and PCA in the training and testing sets.

### Functional analysis and GSEA

**Figure 8:**
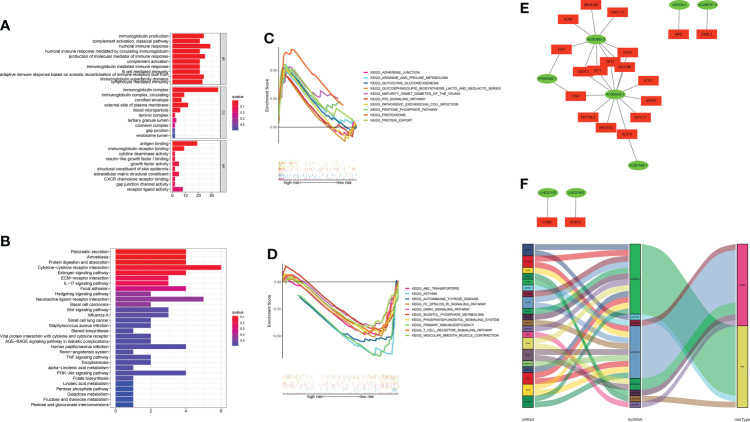
Functional enrichment analysis and mRNA–lncRNA co-expression network. **(A, B)** GO analysis and KEGG analysis. **(C, D)** GSEA of the top 10 pathways significantly enriched in the high- and low-risk groups. **(E)** The network diagram between GT-related lncRNAs and their target mRNAs. **(F)** Sankey diagram of GT-related mRNAs and lncRNAs.

A functional enrichment analysis for the underlying functional and molecular mechanisms of the GT-based model was conducted. GO analysis revealed that these lncRNAs were significantly enriched to 48 biological process (BP) terms, five cell component (CC) terms, and two molecular function (MF) terms. The top three GO terms for BP were the humoral immune response, production of molecular mediator of immune response, and immunoglobulin production. The top GO terms for CC and MF were the immunoglobulin complex and antigen binding, respectively. **Figure 8A** displays the top 10 of GO-BP, all of GO-CC, and all of GO-MF terms. KEGG analysis revealed that cytokine–cytokine receptor interaction, neuroactive ligand–receptor interaction, and pancreatic secretion were the top three significantly enriched pathways. The top 10 KEGG pathways are shown in **Figure 8B**. In order to better investigate discrepancies in biological functions between the two risk groups, we explored the enrichment of KEGG pathways by GSEA software. As shown in **Figures 8C, D**, pathways such as proteasome were enriched in the high-risk group significantly, while in the low-risk group, the significantly enriched pathways were asthma and primary immunodeficiency.

### The mRNA–lncRNA co-expression network

Considering the direct regulatory role of lncRNAs and mRNAs in LUAD, we constructed the mRNA–lncRNA co-expression network and visualized the correlations utilizing Cytoscape. The regulatory network consists of 28 pairs of lncRNA–mRNAs reaching the threshold, of which 20 mRNAs are significantly correlated with the lncRNAs (**Figure 8E**). The co-expressed network was also depicted in the Sankey diagram, which showed that AC005034.3 and AL603839.3 might be the major components (**Figure 8F**).

### TME and immunotherapy

**Figure 9:**
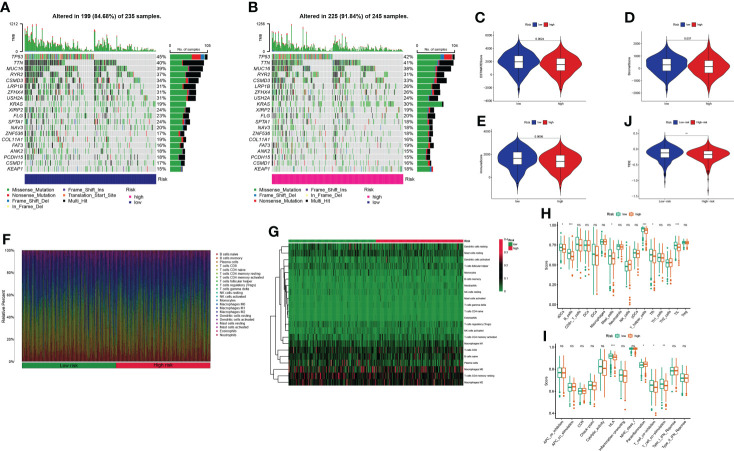
Estimation of the tumor immune microenvironment and cancer immunotherapy response in TCGA entire set; ^*^P< 0.05, ^**^P< 0.01, ^***^P< 0.001, and ^ns^P > 0.05. **(A, B)** Waterfall plot displays mutation information of the genes with high mutation frequencies in the low- and high-risk groups. **(C–E)** Violin plot of the difference in estimate score, stromal score, and immune score between two risk groups. **(F–G)** Bar chart and heatmap plot of the proportions of 22 tumor-infiltrating immune cell types. **(H–I)** The relationship of the risk score with immune cell infiltration and the immune response. **(J)** TIDE prediction difference in the high- and low-risk patients.

Mutation data were processed and analyzed by the R package “maftools,” stratified according to the variant effect predictor, and the waterfall map was used to display the mutation information of high-frequency mutation genes in different risk groups. The top 20 driver genes are shown in **Figures 9A** **, B**. Considering the role of GT-related lncRNAs in the tumor microenvironment of LUAD, the estimate score, stromal score, and immune score of each patient were calculated through the ESTIMATE algorithm. **Figures 9C–E** show that these scores were higher in the low-risk group. We used a bar chart and heatmap plot to show the proportions of 22 tumor-infiltrating immune cell types in the two risk groups (**Figures 9F, G**). It was worth noting that CD4 naive T cells did not express in both risk groups with the screening of CIBERSORT. Six immune cells, such as B cells, and four immune functions, such as HLA, were more closely related to the high-risk group through comparing the ssGSEA scores (**Figures 9H, I**). Then, the clinical immunotherapy efficacy was estimated by TIDE in different risk groups. The higher the TIDE prediction score, the greater the probability of immune evasion, suggesting that patients were less likely to receive the benefits of immunotherapy. We could conclude that immunotherapy has a greater response in the high-risk group, indicating that our GT-based classification index could be used as an indicator to predict TIDE (**Figure 9J**).

### Candidate drugs

**Figure 10:**
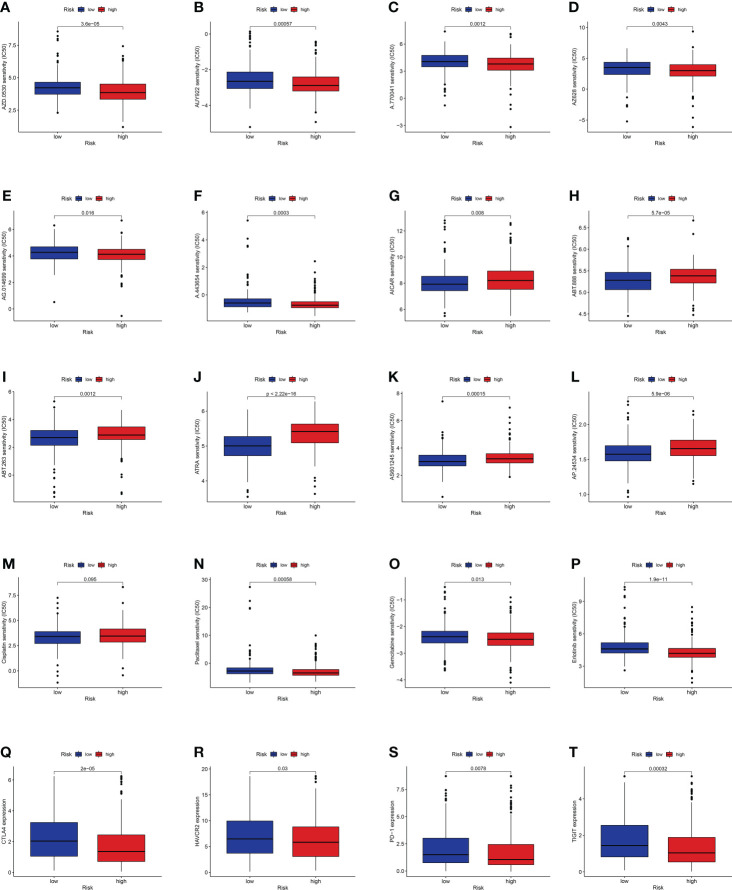
**(A–L)** Identification of novel candidate drugs targeting the GT-related lncRNA model. **(M–P)** Investigation of antitumor drug sensitivity-targeting signature. **(Q–T)** Expression levels of CTLA4, HAVCR2, PD-1, and TIGIT in the high- and low-risk groups.

Considering the discrepancies in the immune microenvironment between the two risk groups, we hypothesized that the two groups may respond differently to drugs. Then, we estimated the treatment response based on IC_50_ of each sample in the GDSC database using the pRophetic algorithm for potential drugs targeting our model (**Figure 10**). The IC_50_ values of A.443654, A.770041, AZ628, AUY922, AG.014699, and AZD.0530 were significantly lower in the high-risk group, suggesting that patients in the high-risk group respond to these drugs better (**Figures 10A–F**). Similarly, the IC_50_ values of ABT.263, AP.24534, ABT.888, AICAR, AS601245, and ATRA were significantly higher in the high-risk group, suggesting that patients in the low-risk group were more likely to benefit from them (**Figures 10G–L**). Additionally, we counted the IC_50_ of common anti-lung cancer drugs in two subgroups. Patients in the low-risk groups were related with a higher IC_50_ of paclitaxel, gemcitabine, and erlotinib, which indicated that the risk model served as a promising predictor of antitumor drug sensitivity (**Figures 10M–P**). In addition, with immune checkpoint inhibitors (ICIs) having been applied in the treatment of LUAD and other cancers, we further explored the differences in ICI-related biomarker expression among two subgroups. The results presented that the levels of CTLA4, HAVCR2, PD-1, and TIGIT were higher in the low-risk group (**Figures 10Q–T**).

### The validation by intro experiments

**Figure 11:**
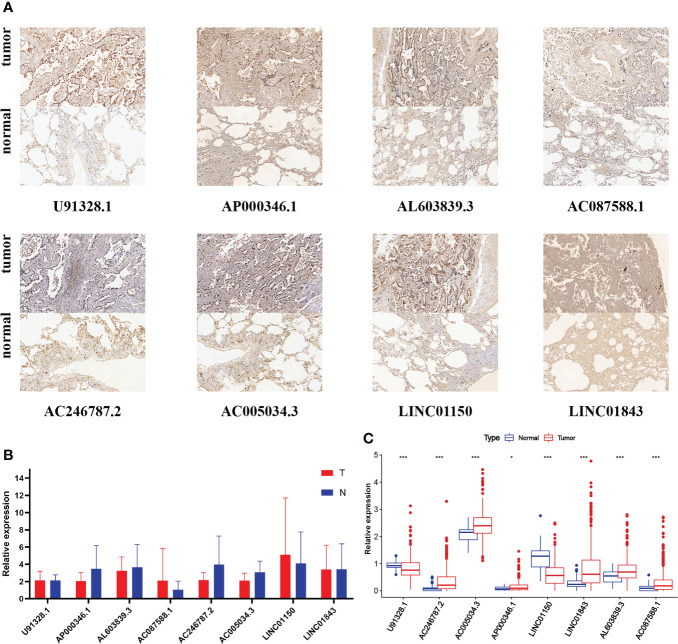
**(A)** CISH of the eight GT-related lncRNAs in LUAD tissues and corresponding normal tissues. **(B)** Expression levels of eight GT-related lncRNAs in LUAD tissues and corresponding normal tissues by RT-qPCR. **(C)** Expression levels of eight GT-related lncRNAs among 535 LUAD and 59 normal tissues based on TCGA database; *P< 0.05, ***P< 0.001.

Considering that the functions of lncRNAs were tightly associated with subcellular distribution, we first used bioinformatic analysis to predict the location by lncLocator 2.0. Recent statistics on lncATLAs have shown that lncRNAs may be located differently in different cell lines. Thus, we specifically chose the A549 cell line for prediction because the object of our study was LUAD patients and the AUROC value of lncLocator 2.0 in the A549 cell line was 0.8499. The CNRCI value indicated the logarithmic ratio of concentration in the cytoplasm to the nucleus, and a value greater or less than 1 indicated a higher concentration of lncRNAs in the cytoplasm or nucleus. It was predicted that half of the eight lncRNAs (AC246787.2, AC005034.3, LINC01150, LINC01843) were located in the nucleus, and the other half (U91328.1, AP000346.1, AL603839.3, AC087588.1) in the cytoplasm (**Supplementary Table S7**). The predictions above were further confirmed by CISH. **Figure 11A** shows that the expression of the eight lncRNAs in LUAD tissues was higher than that in paracancerous tissues. However, almost all lncRNAs were mainly distributed in the nuclei of LUAD tissues, except LINC01843. The RT-qPCR method was also used to detect the expression of the eight lncRNAs in 10 pairs of LUAD and paracancerous tissues. As shown in **Figure 11B**, we did not find any difference between cancer and paracancerous tissues due to the small number of tissues. We further verified the differences in the expression of these eight lncRNAs between LUAD samples and standard tissue samples based on TCGA database, and the differences are displayed in **Figure 11C**.

## Discussion

LUAD, the most common subtype of lung cancer, is seriously harmful to people’s health. Its occurrence, development, diagnosis, and treatment have increasingly become the focus of research (27). Although the popularity of low-dose chest CT helped clinicians detect a considerable number of early lung cancers in time, the OS of LUAD patients was still low, which may be attributed to the lack of timely diagnosis and effective treatment (28). Recently, a considerable number of studies have focused on constructing prognostic models with non-coding RNA to predict patient survival and immunotherapy response in numerous cancer fields (29). Shen et al. reported that lncRNA GHET1 may be a prognostic biomarker and molecular target of NSCLC and provide an underlying therapeutic target (30). LncRNA HUMT was significantly upregulated in lymph node-invasive cells of triple-negative breast cancer, which indicated a poorer clinical outcome (31). Similarly, lncRNA HOTAIR led to tumor metastasis by reprograming the chromatin state, which suggested that lncRNAs possess the potential to become important targets of tumor diagnosis and treatment for their active role in regulating the tumor epigenome (32).

Glycosylation, the most common and complex method of posttranslational modification of proteins, was essential for cell adhesion, stability, and intercellular communication in all eukaryotic cells (17, 33). Glycosylated proteins and other sugar conjugates defined and regulated several critical physiological processes and were also the main components of cells (34). Some factors including heredity, epigenetics, metabolism, and inflammatory and environmental mechanisms could drive the occurrence and development of cancer by promoting glycosylation modifications (35). For example, silencing of epigenetics during tumor progression was the key to elongating O-glycans, which could promote the hypermethylation of the core 1β3-galactosyltransferasespecific molecular chaperone (20, 36, 37). In recent years, the role of glycosyltransferase has attracted a lot of attention in various cancers and has been reported. Glycosyltransferase and O-glycosylation were expressed significantly more highly in breast cancer tissues than in adjacent tissues (38). The expression of glycosyltransferase in primary cancer was significantly lower than that in metastatic colon cancer (39, 40). Additionally, various studies have shown that there is a strong correlation between glycosylation level and tumor malignant degree in endometrial carcinoma, prostate cancer, liver cancer, and gastric cancer, suggesting that abnormal levels of glycosylation may be involved in tumor progression (41–44).

Glycosylation changes in tumor cells usually occur in the early tumor stages, and some tumor-related glycans have been shown to be expressed in precursors of different types of cancer, making them powerful biomarkers for early diagnosis (18, 45). Wang et al. found a GT-related gene marker that could predict the OS of ovarian cancer (46). However, studies on GT-related lncRNAs in tumor prognosis and treatment are still limited. GT-related lncRNAs in LUAD deserve more attention. As far as we know, our study is the first to combine glycosylation and lncRNA for establishing a LUAD prognostic model, which could help predict the prognosis and evaluate the sensitivity of immunotherapy.

In our study, we first constructed the eight-GT-related lncRNA risk model using the methods described above and the excellent AUC values demonstrated its excellent performance in predicting the OS of LUAD patients. After confirming that our model was reliable to predict the prognosis, we established a nomogram including clinical characteristics of LUAD patients for testing the ability of our model in clinical work, and PCA and t-SNE were also conducted to verify the accuracy. The signature was further evaluated in various clinical characters, including immune cell infiltration, TME, and IC_50_ of candidate drugs. We also carried out functional enrichment analysis to explore the role of these lncRNAs at the molecular level. Finally, *in vitro* experiments including CISH and PCR were conducted to validate the model.

The eight hub lncRNAs comprised three risk factors (AC005034.3, LINC01843, AC087588.1) and five protective factors (U91328.1, AC246787.2, AP000346.1, LINC01150, AL603839.3). Li et al. found that LINC01843, as one of seven immune-related lncRNAs, participated in the construction of the LUAD prognostic model. He et al. also found that LINC01150 and LINC01843 were involved in the construction of the LUAD prognostic model, similar to two of the seven immune-related lncRNAs. Considering that these two lncRNAs are also GT-related lncRNA, this may suggest that there may be a link between glycosylation and immunity in LUAD. However, most of them (AC005034.3, AC087588.1, U91328.1, AC246787.2, AP000346.1, AL603839.3) have not been reported in any cancers before. They had the potential to become LUAD prognostic markers, which deserve further exploration and study.

There have been numerous detailed studies on tumor glycobiology while how tumor glycosylation affects the activity of immune cells in the tumor microenvironment is still a new research orientation. Some reviews have pointed out that tumor glycosylation could be explored as a new index for tumor diagnosis and prognosis, which may be related to immune infiltration scores. Therefore, we also focused on TME and immunotherapy response in this study using the GT-related lncRNA model. Firstly, immune cells and stromal cells were the main components of TME, and immune and stromal scores were associated with clinic characteristics as well as prognosis in LUAD. Thus, estimate score, stromal score, and immune score were calculated through the ESTIMATE algorithm for each patient, and these scores were lower in high-risk groups. By comparing the ssGSEA scores of immune cells and immune function, we also found that people associated with these six immune cells and four immune functions were at a higher risk. This finding may be helpful for immunotherapy in high-risk groups. Numerous studies have used the TIDE prediction score which was developed as a computational framework to predict the efficacy of immunotherapy, and its prediction performance has been verified comprehensively. In this study, the prediction using TEDE showed that immunotherapy was more effective for patients in high-risk groups, indicating that our prognostic model could be utilized as a marker in immunotherapy. Finally, we also predicted the drug sensitivity targeting our prognostic model. Based on the results above, we could speculate that our prognostic signature may serve as a reliable immune biomarker for survival prediction and oncotherapy.

Functional enrichment analysis was utilized for the underlying molecular mechanisms of our model. Besides, we analyzed the two risk groups in the KEGG pathway for investigating their discrepancies in biological functions by GSEA software. We found that proteasome and other pathways were significantly enriched in high-risk groups, while asthma and primary immunodeficiency were significantly enriched in low-risk groups. Considering the role of the interaction between lncRNAs and mRNAs in tumor development, we also established the mRNA–lncRNA co-expression network.

Additionally, we did some validation work by *in vitro* experiment. The subcellular localization of lncRNA was closely related to its function. However, the subcellular localization of the eight lncRNAs in LUAD tissues has not been reported, so we combined LncRNAs2.0 and CISH to describe their distribution. It could provide great guiding significance for further exploring the mechanism of these lncRNAs. Through RT-qPCR, we could see the differences in the expression of these eight lncRNAs in 10 pairs of LUAD and paracancerous tissues. However, no statistical difference was found in the expression. This result may be related to the large variance between-sample result of the small number of tissues we used for PCR validation.

We also recognized some limitations and shortcomings of this study. First, our model was validated only in TCGA cohort and its performance would be further verified if it could be validated externally in other clinical datasets with a larger sample size. Second, our experiment methods were not comprehensive. The sample size of the tissue we selected in the validation experiment is insufficient. We could verify the accuracy of our model through *in vivo* experiments, and the biological mechanism of GT-related lncRNAs at the molecular level in LUAD is also worthy of our attention. Thus, in the next study, we will attempt to investigate the role of lncRNAs in LUAD and their interaction with targeting GT-related genes.

In summary, we constructed an eight-GT-related lncRNAs risk model with the ability of being an independent prognostic variable for LUAD patients. Our model may provide some new insights into the involvement of glycosylation in the formation of cancer and guide precise treatments. We hope that our study could help identify novel biomarkers for subsequent studies to clarify the specific process and potential mechanism of lncRNAs regulated by glycosylation.

## Data availability statement

The original contributions presented in the study are included in the article/**Supplementary Material**. Further inquiries can be directed to the corresponding authors.

## Ethics statement

The studies involving human participants were reviewed and approved by Ethics Committee of Jiangsu Province People’s Hospital and the First Affiliated Hospital of Nanjing Medical University. The ethics committee waived the requirement of written informed consent for participation.

## Author contributions

CB, XS, and JH designed the study and prepared the manuscript. WZ and GM contributed to data acquisition and analysis. KW optimized the *in vitro* experiments. LC, YX, and JW revised the manuscript. CB, XS, and JH contributed equally to this work and shared the first authorship. All authors contributed to the article and approved the submitted version.

## Funding

This work was supported by the Jiangsu Province Natural Science Foundation (BK20201492) and Key Medical Research Project of Jiangsu Provincial Health Commission (K2019002).

## Conflict of interest

The authors declare that the research was conducted in the absence of any commercial or financial relationships that could be construed as a potential conflict of interest.

## Publisher’s note

All claims expressed in this article are solely those of the authors and do not necessarily represent those of their affiliated organizations, or those of the publisher, the editors and the reviewers. Any product that may be evaluated in this article, or claim that may be made by its manufacturer, is not guaranteed or endorsed by the publisher.

## Supplementary material

Supplementary Figure 1 The 184 GT-related lncRNAs significantly related to OS

Supplementary Table 1 The 14056 lncRNAs from the TCGA database

Supplementary Table 2 The 257 GT-related genes from the GSEA database

Supplementary Table 3 The baseline characteristics of LUAD samples

Supplementary Table 4 The probe sequences of CISH

Supplementary Table 5 The primer sequences of PCR

Supplementary Table 6 The 8 lncRNAs for constructing the prognostic model

Supplementary Table 7 The prediction result of lncLocator 2.0

Supplementary Methods Detailed steps of the CISH experiment
