# Supplementary figures and images for: A novel glycosyltransferase-related lncRNA signature correlates with lung adenocarcinoma prognosis
Source: Front Oncol. 2022 Aug 18;12:950783. doi: 10.3389/fonc.2022.950783 (PMC9434379; doi:10.3389/fonc.2022.950783)

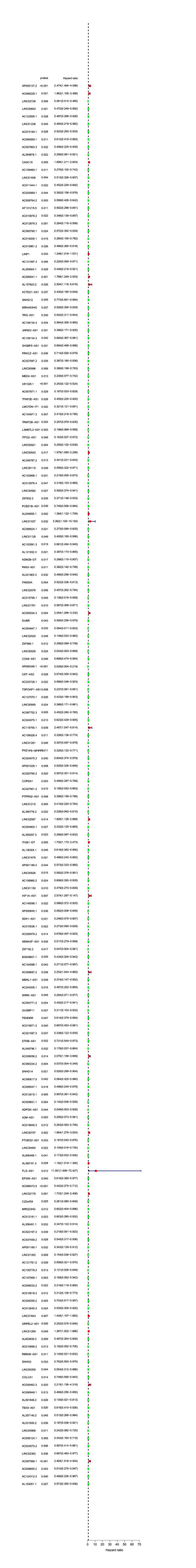

Supplement: Supplementary Figure 1 — The 184 GT-related lncRNAs significantly related to OS [file Image_1.tif]
