# Supplementary material for: A novel glycosyltransferase-related lncRNA signature correlates with lung adenocarcinoma prognosis
Source: Front Oncol. 2022 Aug 18;12:950783. doi: 10.3389/fonc.2022.950783 (PMC9434379; doi:10.3389/fonc.2022.950783)
Supplement: Supplementary Table 3 — The baseline characteristics of LUAD samples [file Table_3.docx]

summary of patients’ characteristics

| Characteristics | Type | Entire set | Training set | Testing set | P value |
| --- | --- | --- | --- | --- | --- |
| Age | <=65 | 231(47.14%) | 131(53.25%) | 100(40.98%) | 0.0133 |
|  | >65 | 249(50.82%) | 112(45.53%) | 137(56.15%) |  |
|  | unknow | 10(2.04%) | 3(1.22%) | 7(2.87%) |  |
| Gender | FEMALE | 262(53.47%) | 137(55.69%) | 125(51.23%) | 0.3684 |
|  | MALE | 228(46.53%) | 109(44.31%) | 119(48.77%) |  |
| Stage | Stage I-II | 378(77.1%) | 186(75.6%) | 192(78.7%) | 0.4923 |
|  | Stage III-IV | 104(21.2%) | 56(22.8%) | 48(19.7%) |  |
|  | unknow | 8(1.7%) | 4(1.6%) | 4(1.6%) |  |
| T | T1-2 | 426(86.9%) | 213(86.6%) | 213(86.6%) | 0.932 |
|  | T3-4 | 61(12.5%) | 32(13.0%) | 29(11.9%) |  |
|  | unknow | 3(0.6%) | 1(0.41%) | 2(0.82%) |  |
| M | M0 | 324(66.1%) | 156(63.4%) | 168(68.9%) | 0.4523 |
|  | M1 | 24(4.9%) | 14(5.7%) | 10(4.1%) |  |
|  | unknow | 142(29.0%) | 76(30.9%) | 66(27.0%) |  |
| N | N0 | 317(64.7%) | 164(66.7%) | 153(62.7%) | 0.3239 |
|  | N1-3 | 162(33.1%) | 78(31.7%) | 84(34.4) |  |
|  | unknow | 11(2.22%) | 4(1.6%) | 7(2.9%) |  |
