# Supplementary material for: A novel glycosyltransferase-related lncRNA signature correlates with lung adenocarcinoma prognosis
Source: Front Oncol. 2022 Aug 18;12:950783. doi: 10.3389/fonc.2022.950783 (PMC9434379; doi:10.3389/fonc.2022.950783)
Supplement: Supplementary Methods — Detailed steps of the CISH experiment [file DataSheet_1.docx]

**The steps of the CISH experiment**

1. **Organization fixed**: Take out the organization, wash clean, then immediately put in the fixed fluid (DEPC) to fix 12h.

2. **Dehydration**: The tissue is dehydrated by gradient alcohol.

3. **Section**: The paraffin is sliced and roasted for 2 hours in the 62℃ oven.

4. **Dewaxing and dehydration**: Soak sections in 2 changes of Dewaxing Transparent Liquid, 15 minutes each. Dehydrate in 2 changes of pure ethanol for 5 minutes each. Wash in DEPC dilution.

5. **Digestion**: According to the tissue fixation time, the slices are boiled in the retrieval solution for 15 minutes and naturally cooled. Mark the objective tissue with a liquid blocker pen, according to the characteristics of the tissues. Add proteinase K(20ug/ml) working solution to cover the objectives and incubate at 37℃ for 20 min. Washing with pure water, then wash three times with PBS in a Rocker device, 5 min each.

6. **Block endogenous peroxidase**: Add 3% methanol-H_2_O_2_, incubate in dark at room temperature for 15min. Wash slides in PBS three times for 5 min each, with gentle agitation and protection from light.

7. **Pre-hybridization**: Add Pre-hybridization solution to each section and incubate for 1 hour at 37℃.

8. **Hybridization**: Discard the pre-hybridization solution, add the probe hybridization solution and incubate the section in a humidity chamber and hybridize overnight at 42℃.

9. **Washing**: Remove the hybridization solution with SSC.

10. **Imaging oligo (DIG**): Add the hybridization solution containing Imaging Oligo (DIG), dilution ratio 1:400, 42℃ FOR 3 hours. Wash sections with SSC.

11. **Blocking**: Add blocking solution (Rabbit serum) to the section and incubate at room temperature for 30 min.

12. **Add the mouse anti-digoxigenin-labeled peroxidase (anti-DIG-HRP)**: Remove the blocking solution and add anti-DIG-HRP. Incubate at 37℃ for 50 min. Wash sections in PBS four times for 5 min each.

13. **DAB developing**: Dry sections slightly, and add fresh prepared DAB chromogenic reagent to marked tissue. Manage reaction time by observing in microscopy until positive expression appears brown-yellow. Then stop developing reaction by washing in running tap water.

14. **Counterstain in the nucleus**: Counterstain with Hematoxylin staining solution for 3 min and wash in tap water. Treat with the 1% acid alcohol differentiation solution for a few seconds, and wash in running tap water. Back to blue by bluing solution, wash in running tap water.

15. **Dehydration and mounting**: Dehydrate successively in gradient ethanol of 75%, 85%, and 2 changes of pure ethanol, respectively, 6 min each. Clear in Dewaxing Transparent Liquid for 6 min and mount with SweSuper Clean BioMount Medium.
